# The Free Energy Barrier for Arginine Gating Charge Translation Is Altered by Mutations in the Voltage Sensor Domain

**DOI:** 10.1371/journal.pone.0045880

**Published:** 2012-10-19

**Authors:** Christine S. Schwaiger, Sara I. Börjesson, Berk Hess, Björn Wallner, Fredrik Elinder, Erik Lindahl

**Affiliations:** 1 Science for Life Laboratory, Solna, Sweden; 2 Theoretical and Computational Biophysics, Department of Theoretical Physics, Royal Institute of Technology, Stockholm, Sweden; 3 Division of Cell Biology, Department of Clinical and Experimental Medicine, Linköping University, Linköping, Sweden; 4 Department of Physics, Chemistry and Biology and Swedish e-Science Research Center, Linköping University, Linköping, Sweden; 5 Center for Biomembrane Research, Department of Biochemistry & Biophysics, Stockholm University, Stockholm, Sweden; Sackler Medical School, Tel Aviv University, Israel

## Abstract

The gating of voltage-gated ion channels is controlled by the arginine-rich S4 helix of the voltage-sensor domain moving in response to an external potential. Recent studies have suggested that S4 moves in three to four steps to open the conducting pore, thus visiting several intermediate conformations during gating. However, the exact conformational changes are not known in detail. For instance, it has been suggested that there is a local rotation in the helix corresponding to short segments of a 3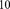-helix moving along S4 during opening and closing. Here, we have explored the energetics of the transition between the fully open state (based on the X-ray structure) and the first intermediate state towards channel closing (C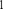), modeled from experimental constraints. We show that conformations within 3 Å of the X-ray structure are obtained in simulations starting from the C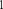 model, and directly observe the previously suggested sliding 3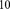-helix region in S4. Through systematic free energy calculations, we show that the C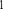 state is a stable intermediate conformation and determine free energy profiles for moving between the states without constraints. Mutations indicate several residues in a narrow hydrophobic band in the voltage sensor contribute to the barrier between the open and C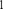 states, with F233 in the S2 helix having the largest influence. Substitution for smaller amino acids reduces the transition cost, while introduction of a larger ring increases it, largely confirming experimental activation shift results. There is a systematic correlation between the local aromatic ring rotation, the arginine barrier crossing, and the corresponding relative free energy. In particular, it appears to be more advantageous for the F233 side chain to rotate towards the extracellular side when arginines cross the hydrophobic region.

## Introduction

Voltage-gated ion channels are membrane proteins that conduct ions, regulated by the electrostatic potential across the membrane. These channels play fundamental roles for instance in the generation and propagation of nerve impulses and in cell homeostasis. They are made up of four homologous domains, each of which contains six transmembrane helices. The first four make up a voltage-sensing domain (VSD), with the helices labeled S1 through S4. The final two helices (S5, S6) line the ion conducting pore together with the same helices from the other three subunits.

Within the VSD, the S4 helix is remarkably atypical for a transmembrane helix since it contains several charged side-chains, primarily arginine but also lysine and occasionally polar substituents. As the electrostatic potential across the membrane is changed, these charges will be subject to large forces that cause S4 to move in the voltage sensor. This in turn initiates a conformation change in the pore domain that opens or closes the channel to control the flow of K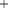 ions from the intracellular to the extracellular side [1]–[6].

Significant progress has been made in understanding the structure and function of voltage-gated ion channels since the early days when it was not even clear that S4 was a transmembrane helix. In particular the X-ray structure of K
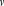
1.2 [7], [8] and later a higher-resolution structure of a K
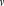
1.2/2.1 chimera (K
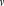
chim) [9] have been instrumental in this process. However, both these structures are for the open state of the channel, and it is only with recent models [10]–[12] as well as functional characterizations [13], [14] and simulations [15] it has become possible to characterize the gating and resting state on atomic level. How are the S4 gating charges stabilized as they effectively move from one side of the bilayer to another, what is the character of the free energy barrier in this process, and are there stable intermediate conformations? What is known is that the gating charges bias the protein to flip between different functional states as a function of the applied voltage. Experimental studies have suggested at least three different functional and conformational states of the voltage sensor [16]; the resting (R) state corresponding to a closed channel when the membrane is polarized, the active (A) state where the channel is open due to depolarization, and the relaxed (AL) state which is an open-inactivated channel reached after prolonged depolarization, which then can require higher and/or longer hyperpolarization to get back to the A and R states. These individual states are separated by free energy barriers [17], and by applying an external potential the relative free energy of the states change, which causes the voltage sensor to make a transition to a different state [18].

In practice, this motion occurs when the additional force on the arginines in S4 cause them to cross the hydrophobic core of the voltage sensor, breaking the salt bridges of the extracellular cluster (with E183/E226) [9]. During this process, each arginine must surmount the free energy barrier that comes from transiently losing the salt bridges, and potentially rotating or distorting the S4 structure [17]. The existence of these intermediate states is well supported experimentally [12].

As a consequence, the activation gating currents can have kinetics in the millisecond up to probably second range kinetics. Functionally, one typically separates between *deactivation* (A to R), *activation* (R to A) and *inactivation* (A to AL). Since the kinetics of these steps differ, there could be important differences either in the barrier, or at or at least differences related to the direction in which the free energy barrier has to be surmounted. The activation and deactivation barriers are likely to be caused by the arginines crossing a hydrophobic zone in the center of the VSD, while the inactivation barrier could also have components related to conformational change (e.g. 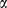
- to 3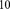-helix transition [16], [17], [19], [20]) in the S4 helix.

The number of charged residues that effectively move across the membrane has been the subject of some debate. A study by Khalili-Araghi *et al.* [20] found the highest-located arginine (R1) in S4 to interact with E183 and E226 in the resting state. This is consistent with the consensus model by Vargas *et al.* [11] and confirmed by a recent gating simulation [15] where three arginines, R2–R4, cross the barrier and R1 is located above the barrier. However, there are experimental indications that R1 too under some circumstances might be able to pass the barrier [12], [14], [21], although this might not be the physiologically most populated down state.

Since there are at least three arginines that cross, there should be intermediate states in the process, with S4 moving down in multiple steps towards the resting state as suggested already by Armstrong [22]. Tao *et al.* [21] suggested a model with five different voltage-sensor states. This would mean that each state indicating one charged residue passing the barrier, i.e. four separate energy barrier steps to overcome. These intermediate states appear to be well supported both by recent models based on metal bridges [12] and simulations of gating [15], although it is unclear whether the intermediate states are also metastable conformations.

The mixture of conserved positive charges in S4 and a hydrophobic region in the center of the VSD supports a switch-like transition between clearly defined arginine interactions above and below the hydrophobic core [9], [23]. We have shown this quantitatively in recent simulations, and suggested it is easier for S4 to make this transition as a 3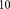-helix [17] based on a somewhat extreme model where the entire segment was forced into 3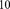 helix.

**Figure 1:**
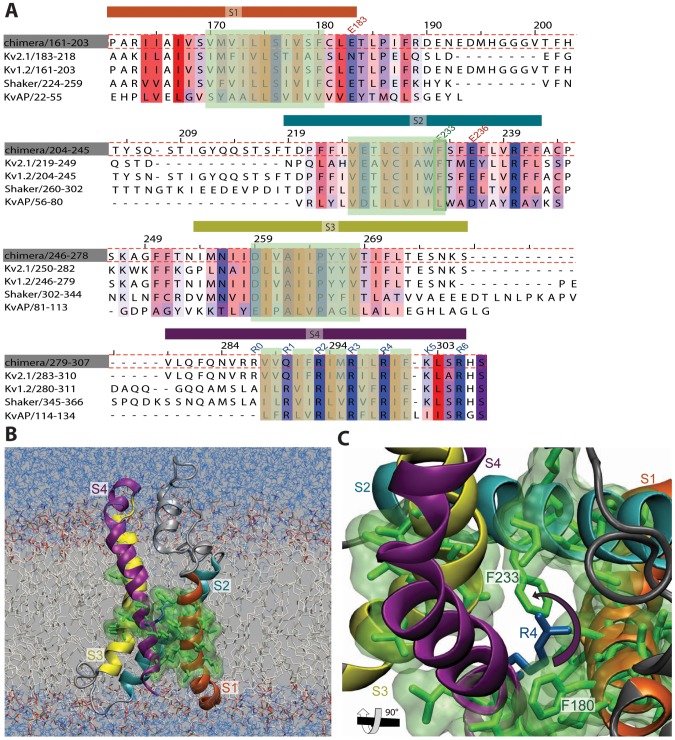
The hydrophobic core of the VSD. *(A)* Sequence alignment of the VSD, visualized with JalView. Residues are colored according to Kyte & Doolittle [50] with blue being hydrophilic and red hydrophobic. Only conserved residues (above 30%) are colored, and the hydrophobic core marked in green overlay. *(B)* Complete simulation system of the open state of the K
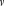
1.2/2.1 chimera, with VSD hydrophobic residues shown in green. (*C*) The hydrophobic core observed from the extracellular side without lipids and water; F233 points into the cavity and might block it.

While there are several residues in the core of the VSD that might affect the barrier (Fig. 1), Long *et al.* suggested the main candidate to be F233 (F290 in Shaker) in the S2 helix, which is almost universally conserved in voltage-gated potassium channels [9]. Tao *et al.* have conducted experiments on F233 mutations [21]. Only F233Y and F233W produced similar results to wild-type Shaker, but they were also able to get functional channels with a non-aromatic cyclohexylalanine side-chain, which suggests a rigid ring is important, but not aromaticity. This appears to be confirmed by our recent simulations that found F233 to act as a structural lock [17] as well as the clear separator for different states [12]. In contrast, Upadhyay *et al.* [24] found F233L to behave almost similar to the wild-type, while it produced a shift of −40–50 mV in the hands of Tao *et al.*. Campos and co-workers [13] found I230C to form a proton pore, which indicates this residue too contributes to the hydrophobic plug. Lacroix and Bezanilla [25] used gating current measurements of several F233 mutants to determine that a hydrophobic residue in position only controls the transfer of R4, the lowest gating charge, while the movement of R1–R3 is not affected. However the nature of the interaction between F233 and the gating charges remains unclear.

Notably, a substitution of the Phe side-chain to a Trp leads to a pronounced relative open state stabilization and consequently a massive left-shift in G/V (pdb: 3LMN) [21]. The replacement with Trp at this position has striking effects on voltage sensor function by a about 40 mV hyperpolarizing shift in activation [21], raising the possibility of an induced intramolecular cation-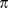 interaction with S4. The Trp substitution was also qualitatively observed to result in significantly slower deactivation. An aromatic side-chain could catalyze the transmembrane passage of S4 charges through cation-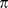 interaction and has been suggested to assist the S4 movement via this mechanism based on refined structural data of the K
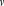
1.2 channel [8]. Pless *et al.* [26] have investigated the possibility that introducing tryptophan at position 233 could form an induced cation-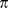 interaction in Shaker and found that the electronegative surface potential of the Trp contributes to a possible such interaction with K5 (Lys302).

To characterize the transition between states, we have created and equilibrated structural models of the first intermediate state during deactivation (one-down; referred to as *closed-1* or *C* 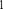) of a voltage sensor based on experimental constraints [12], and used steered molecular dynamics simulations to slowly force it back across the barrier in the direction of the S4 helix axis. We believe this provides a much more natural transition pathway than our (and other) previous constrained studies, and in particular it includes a strict assessment since we can measure how closely the open end state reproduces the known X-ray structure. WIth an unconstrained high-quality reaction coordinate for the S4 translation we have been able to carry out potential of mean force free energy calculations at multiple equilibrium positions rather than nonequilibrium pulling (which leads to hysteresis effects). This has enabled us to perform systematic *in-silico* mutations in the hydrophobic core of the voltage sensor and directly estimate how much mutations affect the free energy barrier for arginine translation. Based on free energy landscapes for the F233 side-chain conformations we also quantify the molecular properties of the lock; the F233 side-chain dynamics appears to be highly important for the kinetics of the gating, and this residue might play the role to keep the the gating kinetics of potassium channels sufficiently slow compared to sodium channels.

## Results

### A reaction coordinate for R4 crossing the hydrophobic core

**Figure 2:**
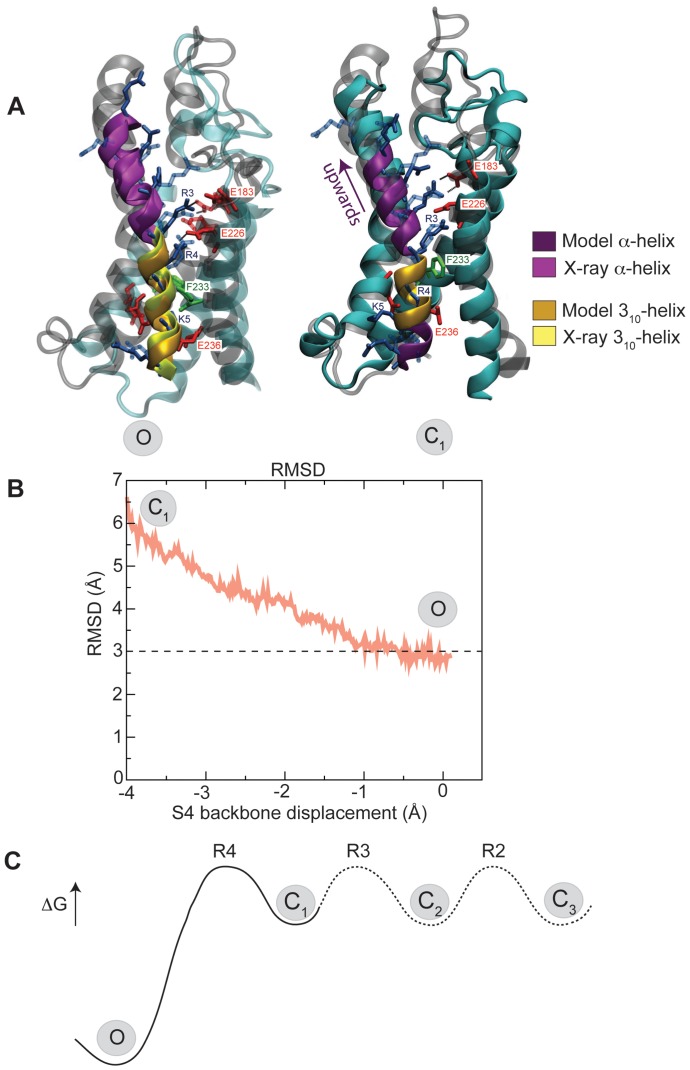
Structure of the VSD before and after steered molecular dynamics. *(A)* Structure of the intermediate C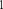 conformation after relaxation, compared to the open X-ray structure (O). In the C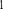 confirmation the R4 arginine side-chain is located below F233, and there is a 3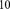-helix region between residues R3 and R4. After forcing S4 (see B) upwards along the helix axis the R4 arginine is located above F233, and the 3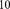-helix has to slide to be located between R4 and K5. This coincides remarkably well with the X-ray VSD structure (O state); the structures are within 3.0 Å RMSD of each other. The X-ray structure is superimposed in gray with arginines in transparent blue. Only the R0 side-chain has a slightly different conformation, which is due to interactions with a lipid. *(B)* C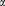 RMSD of the VSD relative to the X-ray structure when starting steered molecular simulation from the C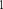 model. Notably, the VSD was *not* specifically pulled towards any coordinates of the O state; the only external component was a force on the charged residues along the S4 helix axis to mimic a potential change. *(C)* Model for the first step of deactivation barrier (O to C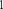 state) caused by R4 moving across the hydrophobic lock. While this study does not characterize the subsequent barriers, they are likely to be smaller, and other simulations too find the first step to be slowest [15]. Having one barrier of greater magnitude could help avoid trapping the S4 helix in intermediate states, while the population of open vs. closed channels will depend on the relative free energies of the O and C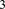 states.

The conformation of the VSD intermediate C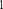 structure after free relaxation for 100 ns is shown in Fig. 2A (left). Helices S1–S3 are virtually identical to the X-ray VSD structure, while S4 has been translated one step down, i.e. the R4 arginine side-chain is located below F233 instead of above it. No restraints were applied during simulation, but the S4 helix spontaneously adopts 3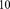-helix structure between residues R3 and R4, exactly where F233 is located, further up in the sequence compared to the X-ray VSD. Limited-range umbrella sampling in both directions confirmed this initial model is very close to the local free energy minimum structure, which appears to confirm that these intermediate states are indeed metastable structures in the free energy sense. The steered molecular dynamics simulation forces S4 to move up, which in turn forces R4 to cross the hydrophobic core and instead adopt a position above F233. The advantage of this limited approach is that it specifically targets a single arginine translation and that the end state of the simulation can be critically compared to experiments. During the simulation, there is a spontaneous change of secondary structure in S4, with the 3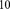-helix region sliding to be most pronounced between R4 and K5. At the end of the upward steered simulation, both the side-chain positions and 3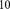-helix location correspond exceptionally well to the features observed in the X-ray structure of the VSD, as illustrated in Fig. 2A (right). Note that S4 is merely pulled along its own helix axis - no information about the X-ray structure is used - but the final simulation conformation reaches an RMSD of only 3.0 Å from the crystal structure (Fig. 2B). This strengthens our confidence in the quality of the obtained reaction coordinate compared e.g. to our own previous qualitative studies where we forced the entire S4 segment into a 3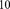-helix as a proof-of-concept in lack of detailed experimental structural information. In particular, these simulations strongly support the model with a conformational shifts between 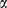-helix and 3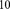-helix, but that this shift occurs through gradual sliding of a constant-length 3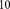-region, rather than a net growth of either structure. In the simulation, the 3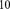-helix region is always located roughly opposite E226 and E236, at the same height as the F233 phenyl ring. Interestingly, this results in a stepwise local rotation of the arginine side-chains around S4 which is very similar to that reported in Fig. 1C of the recent gating simulation of Jensen *et al.* [15], although the secondary structure is not specifically discussed there.

### F233 clearly contributes to the energy barrier

**Figure 3:**
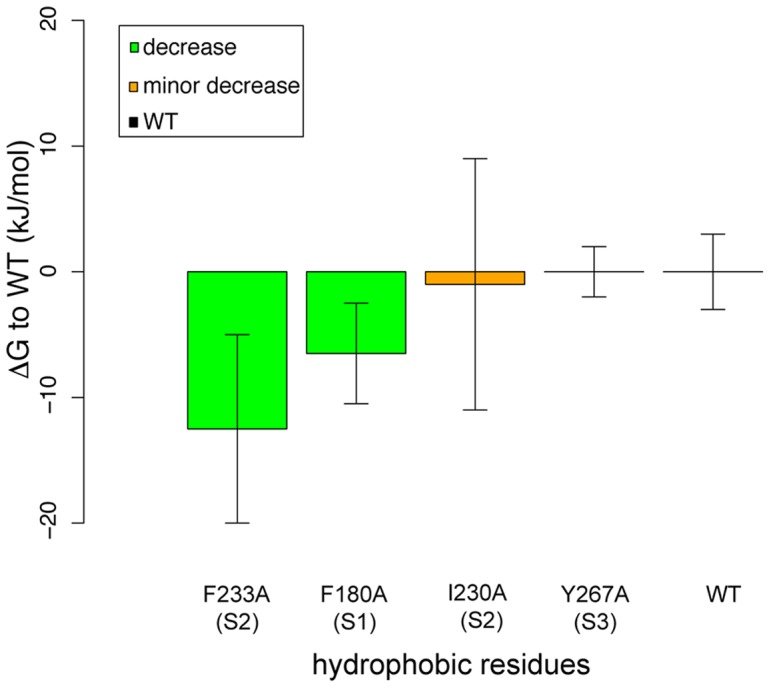
F233A causes the largest reduction in the free energy barrier. Relative difference in the peak value of the free energy barrier during R4 transition for selected hydrophobic residues mutated to alanine, compared to WT. Error bars indicate the standard error of the difference, and the absolute standard error in the calculation for the WT (values for all mutants available in Table 1). The difference is statistically significant both for F180 in the S1 helix and F233 in S2, with the latter showing the greatest shift.

We have previously observed significant free energy barriers to S4 translation [17] at the point where charged side-chains enter the hydrophobic core, but this alone is not sufficient to uniquely identify the source of the free energy cost. To enable this, single-point mutations to alanine were introduced at all 21 residues within 1 nm (Fig. 1) of the hydrophobic core (V170, M171, V172, I173, L174, I175, I177, V178, F180, V225, C229, I230, I231, W232, F233, I260, V261, I263, I264, Y267, V268), and PMF curves calculated as described in the methods. The relative difference in the peak free energy value along the PMF for each residue compared to that of the wild-type is listed in Table 1, and illustrated in Fig. 3 for a subset of positions. Most of the positions (15 out of 21) have little or no effect on the free energy barrier, and there are only two positions where there is a significant decrease when the side-chain is mutated to alanine: F180 and F233. Thus out of all tested residues F233 has the highest effect on the barrier.The shift for F233 is over 10 kJ/mol, more than twice as high as for F180. Both of these phenyl rings face the inside of the cavity, with F233 from helix S2 directly blocking the narrowest part of the VSD pore. In contrast, F180 is located in helix S1 above R4 close to R3 (Fig. 1). The barrier effect from this residue is more likely to come from changes in its interaction with R3 parallel to F233 is interfering with R4. In that case R3 is suspected to rotate slightly as well to overcome F180, but not as much compared to the rotational effect R4 has when crossing F233. In addition, F180 is not as conserved in voltage sensors as F233 is; this position can also contain other hydrophobic residues such as valine or leucine (Fig. 1A). There could be additional contributions from positions such as I230, but these effects are significantly smaller than for the two phenylalanines.

**Table 1:** Relative difference in the peak value of the free energy barrier during R4 transition for all hydrophobic core residues mutated to alanine, compared to the value for WT.

Mutation	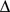 G to WT (kJ/mol)	Effect
F233A (S2)	−12.5 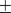 7.5	decrease
F180A (S1)	−6.5 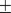 4	decrease
I230A (S2)	−1 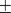 10	minor decrease
Y267A (S3)	0 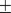 2	no
WT	0 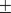 3	-
I231A (S2)	0.5 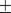 1.5	no
I264A (S3)	0.5 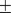 4	no
V170A (S1)	1 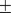 2.5	minor increase
I173A (S1)	2 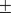 1.5	minor increase
I263A (S3)	2 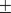 5.5	minor increase
V178A (S1)	2.5 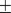 5	minor increase
V268A (S3)	3 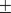 4.5	minor increase
V225A (S2)	3.5 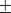 2	minor increase
W232A (S2)	3.5 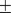 5.5	minor increase
I175A (S1)	4 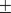 4	minor increase
C229A (S2)	4 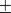 7	minor increase
L174A (S1)	4.5 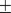 6	minor increase
V172A (S1)	4.5 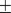 7	minor increase
V261A (S3)	5.5 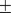 4.5	increase
I260A (S3)	9.5 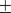 2	increase
M171A (S1)	10.5 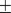 4.5	increase
I177A (S1)	11 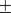 6.5	increase

The standard error for the WT is the absolute standard error. A decrease or increase is defined by the cutoff 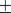 5 kJ/mol. Compare to Fig. 3. In this context, it is worth mentioning the relative differences for Hanatoxin dissociation constants in the closed state of the drk1 VSD, as reported by Swartz [34] and Li-Smerin [35]. While the Hanatoxin data measures the relative stability of O vs C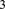 states rather than the kinetics, their study found a 25-fold change upon mutating away the phenylalanine (F274 in drk1), which corresponds to a free energy stabilization of 8 kJ/mol for the open relative to the closed state. The same study reported 13-fold change for the preceding position (6.3 kJ/mol, I273 in drk1), 15-fold change three positions later (6.7 kJ/mol, E277 in drk1), and successively smaller changes for the arginines (R0 4.2 kJ/mol, R2 4 kJ/mol, R3 2.7 kJ/mol, R4 2.1 kJ/mol; R1 in Kv1.2 is a glutamine in drk1). This pattern combined with the limited effect on stability in O vs. C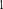 states observed here suggests these mutations primarily destabilize the closed state of the channel.

### Effects of additional mutations on F233

**Figure 4:**
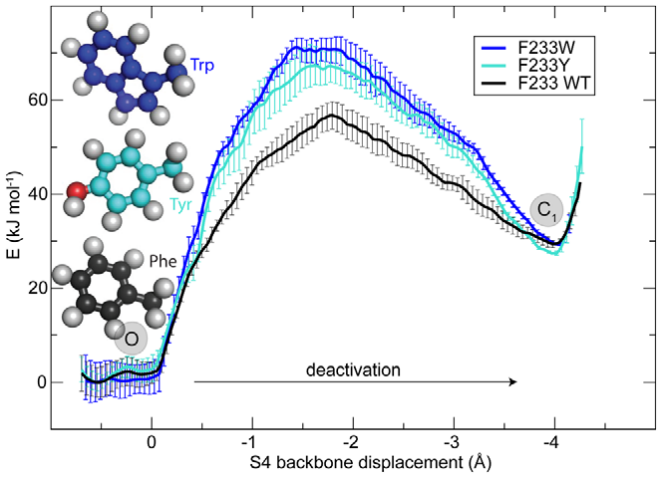
A rigid ring is essential for slow channel deactivation. Both tyrosine and tryptophane increase the cost the S4 transition. The increased bulkiness of these residues makes it harder to move the R4 arginine side-chain across the core region by rotation away the aromatic ring in position 233. The open (O) and first intermediate (C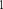) states are indicated. The shape of the barrier for the deactivation fits very nicely to the hypothetical model shown in Fig. 2. The PMF supports that C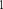 is actually a metastable intermediate state as proposed by Henrion et al. [12].

Given the clear influence on the free energy barrier when the F233 side-chain is removed, it is highly interesting to consider the effect of introducing other amino acids in this position. Fig. 4 displays the PMF profiles for the other available aromatic side-chains, tyrosine and tryptophan, compared to the wild-type (*WT*). Neither of these mutations have any significant structural effect on the voltage sensor. Both F233Y and F233W exhibit higher free energy barriers, with in particular tryptophan making S4 motion significantly harder. Judging from the simulations, the main reason for this barrier is again simple steric hindrance, where all rigid rings provide obstacles, but the double ring of the tryptophan is particularly difficult to rotate away. This would in particular make it harder to deactivate the channel, which appears to agree with experimental results of Tao *et al.* [21] (in contrast to Upadhyay [24]) who found that F233W closing is significantly slowed down due to the mutation. The experiments also found a left voltage shift for F233W, but not F233Y (which instead behaved similar to WT). This is still compatible with the present study since the voltage shift describes the free energy difference between fully open O and fully closed C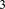 states rather than the O-C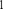 barrier kinetics. (Fig. 2C). Since there is no kinetics data available for F233Y it is not yet possible to directly compare the barrier to experiments for this mutation. The polar groups present both in tyrosine and tryptophane do not appear to play any significant role for their interactions with arginine. On the contrary, despite phenylalanine being more hydrophobic, the fundamental process for all three side-chains is still that the rings have to rotate away, which agrees very well with the observation that a rigid ring at the F233 position is important, but not its aromaticity.

**Figure 5:**
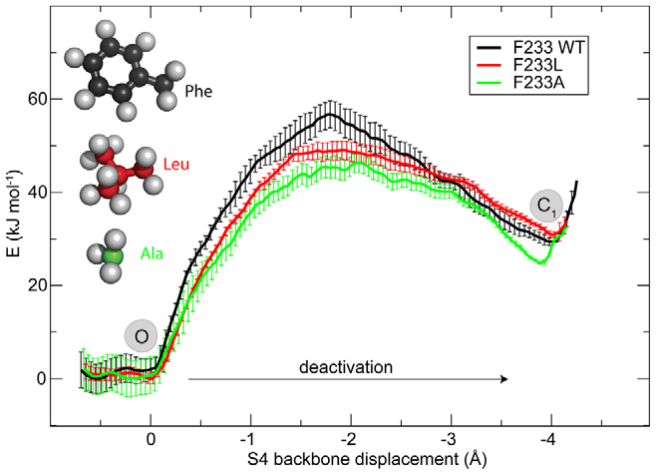
Smaller side-chains decrease the deactivation energy. While leucine is still a large hydrophobic side-chain, it is significantly more flexible than an aromatic ring, which facilitates the translation of R4 across the hydrophobic zone (between O and C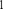 state.

In the steric obstacle model for the barrier, mutations of F233 to smaller residues should have the opposite effect of the large aromatic chains. This is confirmed not only by the F233A mutation, but also by F233L that replaces the phenyl with the most hydrophobic non-ring side-chain. As shown in Fig. 5, the leucine mutation reduces the free energy barrier by roughly 10 kJ/mol, almost as much as the mutation to alanine. Thus, here too the main effect appears to be the bulkiness of the side-chain rather than its hydrophobicity. There is in fact one voltage sensor (K
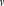
AP) that has a leucine instead of a phenylalanine in the position corresponding to F233, but for virtually all others the phenylalanine appears to be conserved. This could have important implications for gating kinetics, but the relationship between K
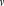
AP other K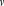 channels is not entirely certain [27].

### F233 conformations during S4 translation

**Figure 6:**
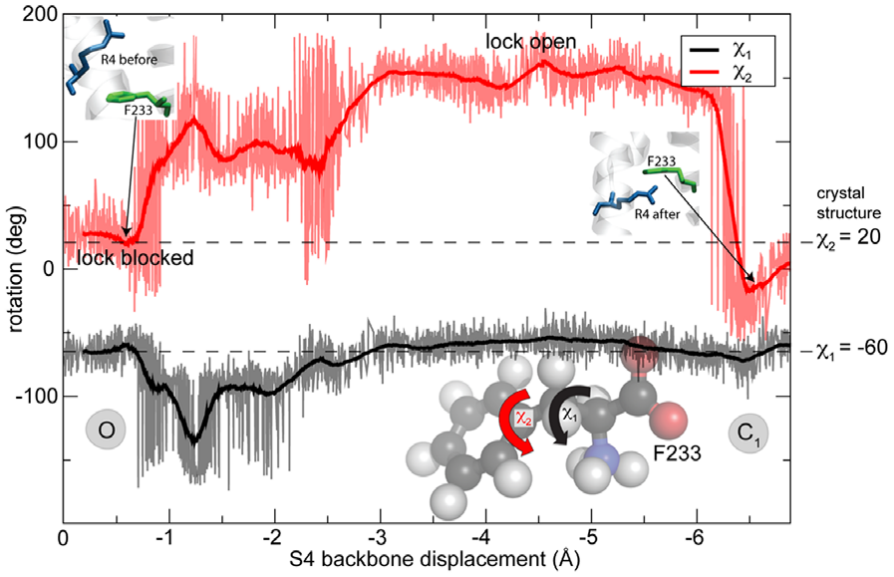
Phenyl ring conformations of R4 crossing the hydrophobic core during deactivation transition. The R4 arginine side-chain motion across the VSD core primarily causes the F233 side-chain to rotate mainly around 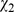 angle in order to open the steric lock formed by the phenyl ring. After passage, the side-chain torsions move back to their initial values close to that of the crystal structure.

Based on the simulations, the phenyl ring appears to alternate between two preferred positions; it either has a largely horizontal orientation (with respect to the membrane frame-of-reference) where it effectively *blocks* the narrowest part of the voltage sensor cavity completely, or it rotates away to a largely vertical orientation when the R4 arginine side-chain needs to pass it. From our earlier simulations where the S4 helix was pulled down slowly from the X-ray structure on microsecond scale [17] it is possible to characterize the phenyl ring conformations in terms of the F233 side-chain torsions 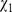 and 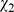 as a function of time (Fig. 6). With the arginine side-chain entering from above (the arginine moves further down to the right in the plot), the ring is initially pushed even harder into a blocked position by R4 (the first 1–1.5 Å of translation), but after a while this lock *opens* by making a jump in the 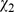 torsion. The 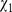 torsion exhibits an initial fluctuation to enable the ring to rotate around 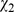 and adopt a largely vertical orientation, but most of the change is observed in 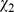. Once R4 has translated down, both 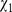 and 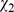 go back to their initial values, and in particular for 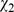 this is a very rapid, switch-like, process. This flipping behavior appears similar to that observed in other studies of aromatic ring motion [28]–[32] that supports the observation that a phenylalanine primarily tends to rotate around the 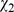 axis. For phenylalanine and tyrosine rings, these motions typically consist of restricted rotational diffusion in 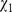 and 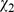 degrees of freedom, combined with 180 ring flips around the symmetry axis in 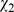 [33].

**Figure 7:**
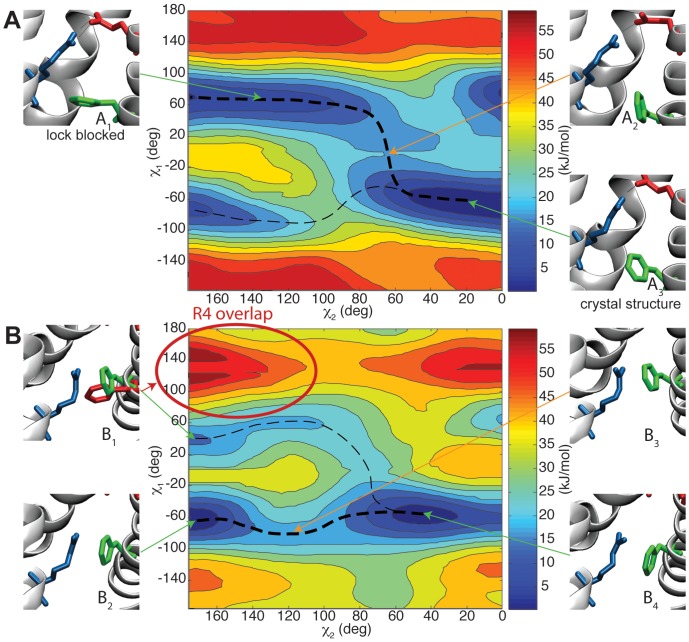
Relative free energy of F233 phenyl ring orientations. 2D PMFplots of relative free energy as a function of side-chain torsions 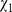 and 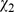, with the R4 arginine (blue) above *(A)* and at the same level (z-wise) as *(B)* F233 (green). E226 is shown in red. There appears to be two possible pathways for F233 to rotate away and open the lock, the first involving a switch-like transition in 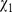, and the second only rotating around 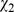. The lowest-barrier path is shown in a bold dashed line. As R4 is located opposite of F233 *(B1)*, the ring's conformational space is restricted by steric clashes (red circle), and it can only rotate around 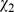 until the arginine has moved further down.

To further study the dynamics of the phenyl ring we performed 2D PMF calculations (see methods) to obtain the relative free energies of its conformations when the R4 side-chain is either above or directly opposite the F233 residue. As illustrated in Fig. 7A, the ring initially has relatively large free energy minima corresponding to the pore-blocking conformations where it has favorable hydrophobic interactions with the VSD core (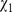 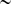 −70, 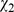 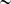 20, conformation A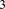 in Fig. 7A). The phenyl ring is pushed by R4 moving down into an even more blocked orientation. This effect has already been characterized in Fig. 6 (initial fluctuations). From these conformations the phenyl ring can rotate away, but this motion is not favorable. As the R4 arginine side-chain is forced down and relocated opposite F233 (Fig. 7B), steric clashes between R4 and the phenyl ring (Fig. 7B, configuration B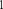) increase the free energy of the pore-blocking state, and eventually make this conformation disadvantageous, at which point the phenyl ring rotates by 100–120 to the vertical open state (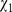 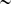 −70, 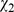 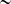 150–170, conformation B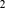 in Fig. 7B). This is followed by a flip-like motion of the arginine across the hydrophobic zone, after which the phenyl ring can rotate back into the lower free energy, pore-blocking, conformation.

## Discussion

There is now increasing evidence - both from experiments and simulation - that the voltage sensor activation/deactivation process is closely coupled to secondary structure alterations in the S4 helix [16], [17], [19], [20] or, equivalently, local rotation of the side-chain adjacent to F233 [15] rather than merely rotation, translation and tilting as a rigid element. The present study shows that our recent models of the intermediate C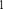 state from experiments is a metastable conformation with a local free energy minimum, and it appears to directly confirm a 3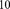-helix region that slides along the S4 sequence as the helix moves from one intermediate conformation to the next. Rather than blindly moving between unknown states, we have shown the end conformation of the present steered molecular dynamics simulations to be remarkably close to the X-ray structure of the chimera voltage sensor, both in terms of side-chain interactions, 3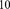-helix contents, and not least a 3 Å RMSD. In relation to our earlier studies qualitatively comparing barriers for constrained 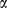-helix vs. 3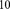-helix translation, this confirms the advantage of 3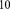-helix structure close to the F233 lock [17] and the gradually changing secondary structure that largely maintains the total fraction of each secondary structure type [12] (thereby removing any net cost for 3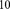-helix formation).

Our potential of mean force calculations largely support the experimental results of Tao [21]. Based on the simulations, it is quite clear that F233 is the functionally single most important residue in the hydrophobic core and provides the main part of the R4 translation barrier. It also suggests that F233 is primarily a steric barrier for the arginine side-chains in the most narrow part of the VSD cavity rather than general hydrophobic effect. Larger aromatic rings increase the barrier despite containing some polar groups, while smaller but almost equally hydrophobic side-chains decrease it. The present PMF-based free energy barriers are also lower than the previous estimates, which makes sense since the S4 helix is now completely unconstrained and allowed to adopt whatever conformation is most advantageous rather than forced to assume either the X-ray or all-3
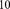
helix structure. However, the agreement is not perfect, in part because the studies are measuring slightly different properties: The simulations presently only cover the path from O through C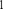 states, which means they can study the deactivation kinetics and also specific stabilization of the O state (relative to C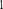). Tao et al. too observed a significant deactivation slowdown for F233W (no kinetics was reported for F233Y), but the left-shifted G/V curves are more related to relative stabilization of O vs. C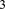 states. In particular, Tao found a left-shift for F233W but not F233Y, and since the present study does not observe any significant differences between O and C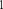 for these mutants that difference would have to occur in the fully closed C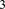 state. Alternatively, it could of course reflect shortcomings in the simulations, for instance different mutants having slight differences in the transition pathway. This is not realistic to sample with the present approach since it would require orders of magnitude more computer resources, but we believe this effect is limited since we do observe clearly lower barriers for some mutants (while they would be higher if we were only distorting the system). However, we believe this question can partly be resolved by considering the mutation data by Swartz [34] and Li-Smerin [35]. These authors measured the relative change in Hanatoxin (known to stabilize closed channels) dissociation constant from voltage sensors of the drk1 channel through alanine scanning, and found large relative changes both for the phenylalanine, the preceding residue, and the residue three positions later. This corresponds to the open state being stabilized (which we do not observe) - or the closed state destabilized - by 6–8 kJ/mol. However, there is also a smaller change in the same direction when mutating the arginines in S4. It is largest for R0 at the top of S4 (5.5-fold, 4.2 kJ/mol), and gradually drops for positions further down in the helix (R1 is glutamine in drk1; R2 5-fold, 4 kJ/mol; R3 3-fold, 2.7 kJ/mol; R4 2.3-fold, 2 kJ/mol). Since R0 & R1 do not interacting much with the VSD in the open state but they are centrally located in the closed one (and the opposite is true for R3 & R4), this pattern strongly suggests the mutations cause a destabilization of the closed (C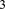) state of the channel, which agrees very well with the results of the present study.

In particular, it needs to be stressed that F233 is not merely a simple steric barrier since a substitutions by smaller hydrophilic residues do not cause omega currents, while it does occur for similar substitutions in R1 or E1. One could speculate that alterations in the salt bridge patterns are particularly important to allow omega currents, while F233's primary role is to regulate the gating kinetics, and the high conservation of this residue could be a key factor in keeping the gating kinetics of voltage-gated potassium channels sufficiently slow compared to sodium channels.

It is highly interesting that all the potential of mean force curves indicate a significantly lower free energy in the open (X-ray) structure to the left in the figures, while the intermediate C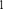 state is clearly higher. Since the present results are based on equilibrium free energy calculations this is unlikely to be an hysteresis effect, and even less so considering we deliberately constructed the reaction coordinate starting from the intermediate state further down; if there were hysteresis effects, we would expect them to overestimate - not underestimate - the relative free energy in the final conformation. These results indicate a hypothetical model for the complete gating process could have a larger step for the first open to C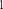 transition, followed by smaller barriers for the remaining steps. Not only would this be necessary to have a low enough free energy difference between the two end states that it is possible to alter it with the externally applied membrane potential, but it is likely also important to avoid having multiple deep minima where the voltage sensor could otherwise be trapped.

There are two limitations in the present model. First, we focus on isolated voltage sensors rather than complete voltage-gated ion channels. This is a slight limitation for more general studies of ion channels since pore conformations likely have some influence at least on the initial stage of deactivation, but it is a good model for the dynamics for isolated voltage-sensing-domains and the core concept of structural transitions in the domain. Second, it has not been possible to include the direct effect of an applied voltage in the present study since we want to study the free energy dependence on conformations during constant external conditions, which makes it difficult to model the O state as depolarized and C1 with some sort of (unknown) intermediate polarization.

Both these are important challenges for further studies, and combination with more experimental results for the kinetics of the voltage sensor activation should help address the questions whether the activation and deactivation processes are mirrors of each other, or have important differences. In particular the F233 properties found in the present study poses interesting questions about the connection between mutations, free energy barriers, and gating kinetics that might be possible to study with simulations. Similarly, a better knowledge of the energetics of the multiple steps should eventually help us understand the important differences between the open vs. open-inactivated conformations observed experimentally [16].

## Materials and Methods

### Experimental derived constraints

The strategy to achieve experimental constraints for the modelling of intermediate states, including the closed-1 (C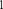) state, has recently been described in Henrion *et al.* [12]. Briefly, residue 325 in helix S3 of the Shaker H4 channel (Acc No NM_167595.3) [36] with the fast, N-type, inactivation removed [37] was probed for possible interactions with residues in a long stretch of S4 (355–369) by constructing double-cysteine mutants (one cysteine in S3 and one in S4). If the pair of cysteines are close to each other (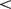
6.5 Å [12]) at some point during channel gating, those cysteines could coordinate a cadmium ion. If so, addition of CdCl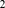 will induce the formation of a metal-ion bridge which alters the behavior of the channel. The effect of cadmium on each mutant was tested electrophysiologically in two-electrode voltage clamped *Xenopus oocytes* expressing the Shaker mutants. One double-cysteine mutant significantly affected by cadmium was 325C/361C showing altered voltage dependence and pronounced current reduction with slow recovery upon cadmium washout. The effect was attributed to the double-cysteine mutation, as the single-cysteine mutants responded differently to cadmium, and was interpreted as a strong metal-ion bridge.

### Isolated voltage sensor simulation model

Helices S1 through S4 from the K
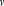
1.2/2.1 chimera (pdb: 2R9R) [9] were used to model an isolated voltage sensor, since this is presently the highest-resolution voltage-gated ion channel available. Many experimental studies on voltage-gated channels [38]–[42] have instead been carried out on the Shaker K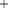 channel, but the two have very high sequence similarity and their voltage sensors should be functionally equivalent. The simulation system was set up as previously described [17], with the modification of the Amber99SB-ILDN force field being used to model the protein [43] (Fig. 1B). All simulations were carried out with Gromacs 4.5.3 [44] using 5 fs time steps with virtual interaction sites for hydrogens.

### Modeling the intermediate C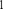 state & transitions

To overcome the lack of structural intermediates, a model of the state where S4 has translated one step down (C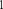 state) was built using Rosetta using the experimental derived constraints in the same way as previously described [12]. The residues starting from S275 at the end of S3 through S307 at the end of S4 were rebuilt using the loop modeling protocol followed by all-atom refinement in Rosetta-membrane [45]. A cadmium-linker constraint between sulfurs of C268 in S3 and C289 in S4 (residues 325 and 361 in Shaker, Henrion *et al.* [12]) was applied in the form of a harmonic constraint centered at 6 Å. No particular secondary structure was enforced in the modelling, but the resulting C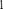 model spontaneously adopted 3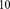-helix between residues 296 and 302, i.e. the region between R3 and K5 facing F233. Comparing with the other models, the 3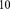-part is conserved in this spatial region, and slides along the amino acid sequence when S4 moves between states. Steered molecular dynamics (SMD) was used to generate a trajectory between the two states by applying a harmonic potential to the center-of-mass of the R0–R6 charged side-chains and the center of mass of S1–S3 helices as described in [17], without constraining any parts of the S4 helix. The steered simulations were started from the C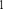 state, pulling upwards in the direction along the helix, which makes it possible to assess to what extent the end state of the SMD simulation coincides with the the original VSD X-ray structure. Additionally, the X-ray structure state was also pulled downwards towards to obtain a transition coordinate for the deactivation process and get information whether C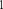 is a metastable state.

### In-silico mutagenesis & potential of mean force calculations

To investigate the barrier, frames spaced 0.02 nm of the S4 center-of-mass apart were selected from the wild-type SMD trajectories, and each hydrophobic residue in the core region of S1 through S3 (S4 is the moving part) mutated to alanine. F233 was additionally mutated to a number of other residues to assess the influence of the phenyl ring and shed light on differences between experiments. All single-point mutations were introduced with PyMol [46] using the mutagenesis plugin. Each mutated system was then energy minimized for 1,000 steps with steepest descent to resolve steric clashes.

Potential of mean force (PMF) curves were calculated by using umbrella sampling on the relative distance between the charged side-chains in S4 (R0–R6) and the reference group formed by helices S1–S3. The umbrella harmonic force constant was set to 10,000 kJ/mol/nm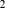, and the reference distance in each window was kept constant during the run. Each umbrella point was sampled for 50 ns, with the first 20 ns (40%) being reserved for relaxation and the remaining 30 ns used for production. Standard errors were estimated by splitting the data into three parts and using jack-knife statistics. PMF curves were calculated from the ensemble of simulations using the Gromacs g_wham program [47] based on the Kumar *et al.* Weighted Histogram Analysis Method [48].

### 2D free energy profile of the phenyl ring rotation

The conformations of the F233 phenyl ring and its interactions with arginine side-chains during S4 translation was investigated through 2D umbrella sampling on the side-chain torsions in F233. This was performed for two different S4 positions, first with the R4 arginine located above F233, and second when directly opposing F233, which corresponds to the peak in the PMF curves. The umbrella term was introduced by modifying parameters of side-chain torsions 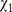 (N-C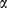-C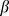-C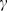), 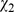 (C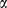-C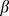-C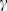-CD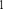) to force the phenyl ring into different conformations. The biasing potential was set to 100 kJ/mol/rad. For 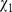, sampling was performed in the range [−180, 180] with a spacing of 10, while 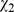 was limited to [0, 180] because of the ring symmetry. Each umbrella simulation was relaxed for 100 ps followed by 1 ns of production. 2D PMF profiles were computed from the simulations using Alan Grossfield's 2D-WHAM program [49].

## References

[1] Yang N, Horn R (1995) Evidence for voltage-dependent s4 movement in sodium channels. Neuron 15: 213–8. 7619524 10.1016/0896-6273(95)90078-0

[2] Yang N, George AL Jr, Horn R (1996) Molecular basis of charge movement in voltage-gated sodium channels. Neuron 16: 113–22. 8562074 10.1016/s0896-6273(00)80028-8

[3] French RJ, Prusak-Sochaczewski E, Zamponi GW, Becker S, Kularatna AS, et al (1996) Interactions between a pore-blocking peptide and the voltage sensor of the sodium channel: an electrostatic approach to channel geometry. Neuron 16: 407–13. 8789955 10.1016/s0896-6273(00)80058-6

[4] Larsson HP, Baker OS, Dhillon DS, Isacoff EY (1996) Transmembrane movement of the Shaker K+ channel S4. Neuron 16: 387–97. 8789953 10.1016/s0896-6273(00)80056-2

[5] Aggarwal SK, MacKinnon R (1996) Contribution of the s4 segment to gating charge in the shaker k+ channel. Neuron 16: 1169–77. 8663993 10.1016/s0896-6273(00)80143-9

[6] Seoh SA, Sigg D, Papazian DM, Bezanilla F (1996) Voltage-sensing residues in the s2 and s4 segments of the shaker k+ channel. Neuron 16: 1159–67. 8663992 10.1016/s0896-6273(00)80142-7

[7] Long SB, Campbell EB, Mackinnon R (2005) Crystal structure of a mammalian voltage-dependent shaker family k+ channel. Science 309: 897–903. 16002581 10.1126/science.1116269

[8] Chen X, Wang Q, Ni F, Ma J (2010) Structure of the full-length shaker potassium channel kv1.2 by normal-mode-based x-ray crystallographic refinement. Proc Natl Acad Sci U S A 107: 11352–7. 20534430 10.1073/pnas.1000142107 PMC2895106

[9] Long SB, Tao X, Campbell EB, MacKinnon R (2007) Atomic structure of a voltage-dependent k+ channel in a lipid membrane-like environment. Nature 450: 376–382. 18004376 10.1038/nature06265

[10] Pathak MM, Yarov-Yarovoy V, Agarwal G, Roux B, Barth P, et al (2007) Closing in on the resting state of the shaker k(+) channel. Neuron 56: 124–40. 17920020 10.1016/j.neuron.2007.09.023

[11] Vargas E, Bezanilla F, Roux B (2011) In search of a consensus model of the resting state of a voltage-sensing domain. Neuron 72: 713–20. 22153369 10.1016/j.neuron.2011.09.024 PMC3268064

[12] Henrion U, Renhorn J, Börjesson SI, Nelson EM, Schwaiger CS, et al (2012) Tracking a complete voltage-sensor cycle with metal-ion bridges. Proc Natl Acad Sci U S A April 25, 2012. doi:101073/pnas1116938109. 10.1073/pnas.1116938109 PMC3365220 22538811

[13] Campos FV, Chanda B, Roux B, Bezanilla F (2007) Two atomic constraints unambiguously position the s4 segment relative to s1 and s2 segments in the closed state of shaker k channel. Proc Natl Acad Sci U S A 104: 7904–7909. 17470814 10.1073/pnas.0702638104 PMC1876545

[14] Lin McA, Hsieh JY, Mock AF, Papazian DM (2011) R1 in the Shaker S4 occupies the gating charge transfer center in the resting state. J Gen Physiol 138: 155–63. 21788609 10.1085/jgp.201110642 PMC3149438

[15] Jensen MØ, Jogini V, Borhani DW, Leffer AE, Dror RO, et al (2012) Mechanism of voltage gating in potassium channels. Science 336: 229–33. 22499946 10.1126/science.1216533

[16] Villalba-Galea CA, Sandtner W, Starace DM, Bezanilla F (2008) S4-based voltage sensors have three major conformations. Proc Natl Acad Sci U S A 105: 17600–17607. 18818307 10.1073/pnas.0807387105 PMC2584729

[17] Schwaiger CS, Bjelkmar P, Hess B, Lindahl E (2011) 310-helix conformation facilitates the transition of a voltage sensor S4 segment toward the down state. Biophysical journal 100: 1446–1454. 21402026 10.1016/j.bpj.2011.02.003 PMC3059565

[18] Bezanilla F (2000) The voltage sensor in voltage-dependent ion channels. Physiological Reviews 80: 555–592. 10747201 10.1152/physrev.2000.80.2.555

[19] Bjelkmar P, Niemela PS, Vattulainen I, Lindahl E (2009) Conformational changes and slow dynamics through microsecond polarized atomistic molecular simulation of an integral kv1.2 ion channel. PLoS Comput Biol 5: e1000289. 19229308 10.1371/journal.pcbi.1000289 PMC2632863

[20] Khalili-Araghi F, Jogini V, Yarov-Yarovoy V, Tajkhorshid E, Roux B, et al (2010) Calculation of the gating charge for the kv1.2 voltage-activated potassium channel. Biophys J 98: 2189–98. 20483327 10.1016/j.bpj.2010.02.056 PMC2872222

[21] Tao X, Lee A, Limapichat W, Dougherty DA, MacKinnon R (2010) A gating charge transfer center in voltage sensors. Science 328: 67–73. 20360102 10.1126/science.1185954 PMC2869078

[22] Armstrong CM (1981) Sodium channels and gating currents. Physiol Rev 61: 644–83. 6265962 10.1152/physrev.1981.61.3.644

[23] Shafrir Y, Durell SR, Guy HR (2008) Models of voltage-dependent conformational changes in nachbac channels. Biophys J 95: 3663–76. 18641074 10.1529/biophysj.108.135335 PMC2553115

[24] Upadhyay SK, Nagarajan P, Mathew MK (2009) Potassium channel opening: a subtle two-step. J Physiol 587: 3851–68. 19528245 10.1113/jphysiol.2009.174730 PMC2746614

[25] Lacroix JJ, Bezanilla F (2011) Control of a final gating charge transition by a hydrophobic residue in the s2 segment of a k+ channel voltage sensor. Proc Natl Acad Sci U S A 108: 6444–9. 21464282 10.1073/pnas.1103397108 PMC3081032

[26] Pless SA, Galpin JD, Niciforovic AP, Ahern CA (2011) Contributions of counter-charge in a potassium channel voltage-sensor domain. Nat Chem Biol 7: 617–23. 21785425 10.1038/nchembio.622 PMC4933587

[27] Cohen BE, Grabe M, Jan LY (2003) Answers and questions from the kvap structures. Neuron 39: 395–400. 12895415 10.1016/s0896-6273(03)00472-0

[28] Kinsey RA, Kintanar A, Oldfield E (1981) Dynamics of amino acid side chains in membrane proteins by high field solid state deuterium nuclear magnetic resonance spectroscopy. phenylalanine, tyrosine, and tryptophan. J Biol Chem 256: 9028–9036. 7263697

[29] Frey MH, DiVerdi JA, Opella SJ (1985) Dynamics of phenylalanine in the solid state by nmr. Journal of the American Chemical Society 107: 7311–7315.

[30] Gall CM, Cross TA, DiVerdi JA, Opella SJ (1982) Protein dynamics by solid-state nmr: aromatic rings of the coat protein in fd bacteriophage. Proc Natl Acad Sci U S A 79: 101–5. 6948294 10.1073/pnas.79.1.101 PMC345669

[31] Levy RM, Sheridan RP (1983) Combined effect of restricted rotational diffusion plus jumps on nuclear magnetic resonance and uorescence probes of aromatic ring motions in proteins. Biophys J 41: 217–21. 6838964 10.1016/S0006-3495(83)84422-1 PMC1329169

[32] Saito H, Ishida M, Yokoi M, Asakura T (1990) Dynamic features of side chains in tyrosine and serine residues of some polypeptides and fibroins in the solid as studied by high-resolution solidstate carbon-13 nmr spectroscopy. Macromolecules 23: 83–88.

[33] Hiraoki T, Kogame A, Nishi N, Tsutsumi A (1998) Deuterium nmr studies on phenyl ring dynamics of poly(-phenylalanine). Journal of Molecular Structure 441: 243–250.

[34] Swartz KJ, MacKinnon R (1997) Mapping the receptor site for hanatoxin, a gating modifier of voltage-dependent k+ channels. Neuron 18: 675–82. 9136775 10.1016/s0896-6273(00)80307-4

[35] Li-Smerin Y, Swartz KJ (2000) Localization and molecular determinants of the Hanatoxin receptors on the voltage-sensing domains of a K(+) channel. The Journal of general physiology 115: 673–684. 10828242 10.1085/jgp.115.6.673 PMC2232886

[36] Kamb A, Tseng-Crank J, Tanouye MA (1988) Multiple products of the drosophila shaker gene may contribute to potassium channel diversity. Neuron 1: 421–30. 3272175 10.1016/0896-6273(88)90192-4

[37] Hoshi T, Zagotta WN, Aldrich RW (1990) Biophysical and molecular mechanisms of shaker potassium channel inactivation. Science 250: 533–8. 2122519 10.1126/science.2122519

[38] Schoppa NE, Sigworth FJ (1998) Activation of Shaker potassium channels. i. characterization of voltage-dependent transitions. J Gen Physiol 111: 271–94. 9450944 10.1085/jgp.111.2.271 PMC2222764

[39] Islas LD, Sigworth FJ (2001) Electrostatics and the gating pore of shaker potassium channels. J Gen Physiol 117: 69–89. 11134232 10.1085/jgp.117.1.69 PMC2232467

[40] Zagotta WN, Hoshi T, Dittman J, Aldrich RW (1994) Shaker potassium channel gating. ii: Transitions in the activation pathway. J Gen Physiol 103: 279–319. 8189207 10.1085/jgp.103.2.279 PMC2216838

[41] Zagotta WN, Hoshi T, Aldrich RW (1994) Shaker potassium channel gating. iii: Evaluation of kinetic models for activation. J Gen Physiol 103: 321–62. 8189208 10.1085/jgp.103.2.321 PMC2216839

[42] Hoshi T, Zagotta WN, Aldrich RW (1994) Shaker potassium channel gating. i: Transitions near the open state. J Gen Physiol 103: 249–78. 8189206 10.1085/jgp.103.2.249 PMC2216835

[43] Lindorff-Larssen K, Piana S, Palmo K, Maragakis P, Klepeis J, et al (2010) Improved side-chain torsion potentials for the amber ff99sb protein force field. Proteins 78: 1950–1958. 20408171 10.1002/prot.22711 PMC2970904

[44] Hess B, Kutzner C, van der Spoel D, Lindahl E (2008) Gromacs 4.0: Algorithms for highly efficient, load-balanced, and scalable molecular simulation. J Chem Theory Comput 4: 435–447. 26620784 10.1021/ct700301q

[45] Barth P, Schonbrun J, Baker D (2007) Toward high-resolution prediction and design of transmembrane helical protein structures. Proc Natl Acad Sci U S A 104: 15682–7. 17905872 10.1073/pnas.0702515104 PMC2000396

[46] Schrödinger, LLC (2010) The pymol molecular graphics system, version 1.2r3pre, http://www.pymol.org.

[47] Hub JS, de Groot BL, van der Spoel D (2010) g wham—a free weighted histogram analysis implementation including robust error and autocorrelation estimates. Journal of Chemical Theory and Computation 6: 3713–3720.

[48] Kumar S, Bouzida D, Swendsen R, Kollman P, Rosenberg J (1992) The weighted histogram analysis method for free-energy calculations on biomolecules .1. the method. Journal of Computational Chemistry 13: 1011–1021.

[49] Grossfield A WHAM: the weighted histogram analysis method (version 2.0.4) website. Available: http://membrane.urmc.rochester.edu/content/wham. Accessed 2011 Nov 11.

[50] Kyte J, Doolittle RF (1982) A simple method for displaying the hydropathic character of a protein. Journal of Molecular Biology 157: 105–132. 7108955 10.1016/0022-2836(82)90515-0

